# Calcifediol During Pregnancy Improves Maternal and Fetal Availability of Vitamin D Compared to Vitamin D3 in Rats and Modifies Fetal Metabolism

**DOI:** 10.3389/fnut.2022.871632

**Published:** 2022-04-12

**Authors:** Antonio Gázquez, María Sánchez-Campillo, Alejandro Barranco, Ricardo Rueda, Jia P. Chan, Matthew J. Kuchan, Elvira Larqué

**Affiliations:** ^1^Department of Animal Physiology, School of Biology, University of Murcia, Murcia, Spain; ^2^Department of Biochemistry and Molecular Biology II, School of Pharmacy, University of Granada, Granada, Spain; ^3^Research and Development Department, Abbott Nutrition SL, Granada, Spain; ^4^Research and Development Department, Abbott Nutrition SL, Singapore, Singapore; ^5^Research and Development Department, Abbott Nutrition SL, Columbus, OH, United States

**Keywords:** availability, calcidiol, calcifediol, pregnancy, vitamin D

## Abstract

The fetus depends on the transplacental transfer of vitamin D. Calcifediol (25-OH-D3) is the vitamin D metabolite that crosses the placenta. Previously, oral 25-OH-D3 improved serum 25-OH-D3 compared to vitamin D3 in non-pregnant subjects, although no studies are available in pregnant women. We evaluated the availability of oral 25-OH-D3 compared to vitamin D3 during pregnancy, as well as, their levels in the fetus and effect on metabolism-related proteins. Twenty female rats per group were fed with 25 μg/kg of diet of vitamin D3 (1,000 UI vitamin D/kg diet) or with 25 μg/kg diet of 25-OH-D3. We analyzed 25-OH-D3 levels in maternal and fetal plasma; protein levels of vitamin D receptor (VDR), fatty acid translocase (FAT), and scavenger-receptor class B type-1 (SR-B1) in both maternal liver and placenta; and protein levels of VDR and Glutamate decarboxylase (GAD67) in fetal brain. 25-OH-D3 doubled the concentration of 25-OH-D3 in both maternal and fetal plasma compared to vitamin D3. In addition, maternal liver VDR, FAT, and SR-BI increased significantly in the 25-OH-D3 group, but no changes were found in the placenta. Interestingly, 25-OH-D3 decreased GAD67 expression in the fetal brain and it also tended to decrease VDR (*P* = 0.086). In conclusion, 25-OH-D3 provided better vitamin D availability for both mother and fetus when administered during pregnancy compared to vitamin D3. No adverse effects on pregnancy outcomes were observed. The effects of 25-OH-D3 on the expression of VDR and GAD67 in fetal brain require further investigation.

## Introduction

Maternal vitamin D insufficiency, during both pregnant and non-pregnant states, is a common issue and a significant problem in public global health (1). Supplementation of food with vitamin D or the use of vitamin D supplements is the most universal strategy to improve vitamin status. Cholecalciferol (vitamin D3) and ergocalciferol (vitamin D2) are the most widely used compounds. While the use of vitamin D3 and vitamin D2 has been supported by historical data and practicality, calcifediol (25-OH-D3) should be evaluated as an alternate oral supplement during pregnancy. Evidence is mounting that it is a more bioavailable form of vitamin D in the non-pregnant state (2, 3).

Oral supplementation with 25-OH-D3 resulted in a more rapid increase in serum 25-OH-D3 compared to oral vitamin D3 in non-pregnant subjects (3). This is consistent with a higher intestinal absorption rate for 25-OH-D3 (4, 5), that may have important advantages when intestinal absorption capacity is decreased due to disease. In addition, as oral 25-OH-D3 is more potent than vitamin D3, lower dosages are needed to achieve desired therapeutic effects (6). There is still no consensus on the vitamin D activity (IU units) conversion factor for 25-OH-D3 and much less is known in the pregnant population (2, 3, 6, 7). Hemodilution may lead to differential responses to vitamin D supplementation between pregnant women. Since some women of reproductive age receive 25-OH-D3 supplementation, it is also important to evaluate the efficiency and risks of such supplementation during pregnancy.

Clinical research investigating the role of vitamin D in human health and disease has relied on the measurement of total 25-OH-D3 in serum or plasma to assess vitamin D status. However, 25-OH-D3 is an inactive form of vitamin D that requires further hydroxylation in the kidneys into 1,25-(OH)2-D3, the active form of vitamin D. However, the active metabolite 1,25-(OH)2-D3 cannot cross the placenta, but 25-OH-D3 readily crosses (8, 9). As the placenta expresses the enzyme 1-α-hydroxylase, it may synthesize 1,25-(OH)2-D3, which seems to play an immunomodulatory role within fetal tissue (9).

The activation of vitamin D receptor (VDR) is heavily dependent on the binding of 1,25-(OH)2-D3 to the receptor (10). The VDR-1,25-(OH)2-D3 complex then translocates into the nucleus to activate DNA transcription. Better understanding of the mechanisms involved in the placental transfer and fetal availability of key nutrients are essential to provide more solid dietary advice to pregnant women.

Fatty acid translocase (FAT/CD36) is a fatty acid carrier placed in the plasma membrane of several tissues, including placenta, and may transport vitamin D and other lipophilic compounds (11). In addition, FAT/CD36 is essential for the very low-density lipoprotein (VLDL) exportation from the liver and its deletion is related to liver steatosis, obesity and non-alcoholic fatty liver disease (12–14). By other hand, scavenger receptor-B1 (SR-B1) is the main receptor of high-density lipoprotein (HDL) in the liver and is pivotal for the uptake of cholesterol from peripheral tissues back into the liver and cholesterol reverse transportation to feces (14). SR-B1 is involved in the cellular uptake of vitamin D (15) but its role in vitamin D tissue storage and the status of vitamin D is currently not known.

In this study, we aimed to compare for the first time the bioavailability of 25-OH-D3 and vitamin D3 administrated during pregnancy. We explored their effects on serum status and on several proteins related to vitamin D transport and metabolism measured in maternal, placenta, and fetal tissues. 25-OH-D3 supplementation is of major interest because it could likely be supplemented at a lower dose than vitamin D3 in order to achieve desirable efficacy in both pregnant women and their babies.

## Materials and Methods

### Animals and Study Design

All procedures were approved by the Institutional Animal Care and Use Committee of the University of Murcia (N° A13180105) and conform to the ARRIVE guidelines for animal research (16). Animals received humane treatment in accordance with the European Union guidelines for the care and use of laboratory animals. Female adult Sprague Dawley rats (7 weeks of age) were supplied by the Animal Laboratory Service of the University of Murcia. Animals were housed individually with *ad libitum* access to food and water in a humidity and temperature-controlled (22 ± 1°C) room on a 12 h light/dark cycle.

**FIGURE 1:**
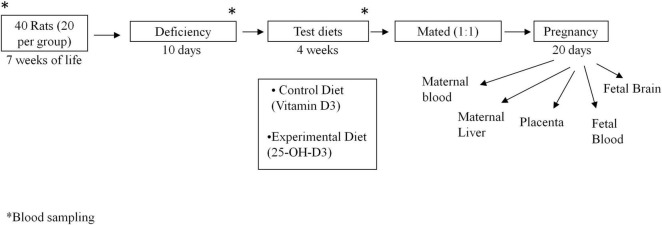
Experimental design. *Blood sample collected at such time of the experiment.

Forty female rats of 7 weeks of age were fed a modified version of AIN-93M diet (17) for 10 days. This modified diet provided by the Abbott Nutrition was deficient in vitamin D, vitamin E and folic acid to ensure similar vitamin basal status in all the animals (Modified AIN-93 Vitamin Mix at 10 g/kg of diet without Vitamin E, Vitamin D, or Folic Acid). Animal weight was recorded every week. After this nutritional deprivation period, the female rats were split into two groups (*n* = 20 each group) and each group of rats were fed a particular test diet for 4 weeks: (1) The control group received commercial AIN-93G diet with 25 μg/kg diet of vitamin D3 at 40 UI/g Vitamin D3 (1,000 UI vitamin D/kg of diet) and (2) the calcifediol experimental group received the AIN-93G diet but with 25 μg/kg of 25-OH-D3 (Merck, Germany) instead of vitamin D3. The commercial AIN-93 G diet used in both control and experimental groups contained vitamin E and folic acid according usual AIN-93G composition. Subsequently, the female rats were mated (1:1) with male rats. Once fecundation took place (by sperm presence in vaginal smear under the microscope), male rats were removed. Female rats were then allocated to appropriate cages and continued to be fed with their assigned test diets throughout the pregnancy. At day 20 of gestation, rats were anesthetized with a mixture of 5 mg ketamine hydrochloride, 0.25 mg chlorobutanol and 1 mg xylazine per 100 g body weight. Maternal blood was extracted by heart puncture and fetal blood by decapitation. Maternal liver, placenta, and fetal brain were also collected (4 placentas and 4 fetal brain were pooled per rat). Additionally, blood samples were also collected from the tail at different stages of the study: (1) at the start of the study (a subset of *n* = 4 animals), (2) after the nutritional deprivation period (another subset of *n* = 4 animals) and (3) after introduction of respective test diets for 4 weeks and right before mating (*n* = 7 controls with Vitamin D3 and *n* = 7 with 25-OH-D3 diet) (Figure 1). Blood was collected in EDTA-coated tubes and centrifugated at 1,400 g for 10 min at 4°C to obtain plasma. Plasma and tissues were frozen in liquid nitrogen and stored at −80°C until analysis.

### Plasma Vitamin D Analyses

25-OH-D3 was analyzed in the plasma of the animals by direct competitive immunoluminometric assay using coated magnetic microparticles in a LIAISON^®^ XL automated analyzer (DiaSorin S.p.A., Saluggia, Italy). The plasma levels of 25-OH-D3 were analyzed using the tail blood samples collected mentioned above. The 25-OH-D3 levels of both maternal and fetal plasma collected at the end the study were also analyzed. Maternal blood was extracted by heart puncture and fetal blood by decapitation at day 20 of gestation.

### Protein Extracts for Western Blotting

Protein extracts were obtained by homogenizing 30 mg of placental tissue, maternal liver or fetal brain in 0.3 mL ice-cold lysis buffer (20 mM Tris-HCl pH 7.5, 150 mM NaCl, 5 mM Na2EDTA, 1 mM EGTA, 1% Triton, 2.5 mM sodium pyrophosphate, 1 mM beta-glycerophosphate, 1 mM Na3VO4, 1 μg/mL leupeptin) from Cell Signaling Technology (MA, United States). Phenylmethanesulfonyl fluoride solution 1 mM was added to the lysis buffer before homogenization (18). Samples were homogenized using a Tissue Lyser LT device (Qiagen Iberia SL, Madrid, Spain). Protein lysates were obtained from the supernatant after 15 min centrifugation at 10.000 g 4°C. Protein concentration was quantified by Bradford assay (19) and samples were stored at −80°C until Western blot analysis.

### Western Blot Analysis

The primary antibodies used were: rabbit monoclonal against FAT/CD36 (Abcam, Cambridge, United Kingdom, Ref: ab17044) 1:250 in maternal liver and 1:200 in placenta; rabbit monoclonal anti-VDR (Abcam, Cambridge, United Kingdom, Ref: ab109234) 1:500 in maternal liver, 1:200 in placenta and 1:400 in fetal brain; rabbit monoclonal antibody against SR-B1 (Abcam, Cambridge, United Kingdom, Ref:ab217318) 1:700; rabbit monoclonal antibody against glutamic acid decarboxylase 67 (GAD67) (Abcam, Cambridge, United Kingdom, Ref: ab108626) 1:700, and mouse monoclonal anti-β-actin (Sigma-Aldrich, MO, United States, Ref:A5316). The secondary antibodies used were anti-mouse (Santa Cruz Biotechnology, TX, United States, sc 516102), anti- rabbit (Santa Cruz Biotechnology, TX, United States, sc-2357) and anti-goat (Sigma-Aldrich, MO, United States, Ref: SAB3700295-1MG) polyclonal antibodies conjugated with horseradish peroxidase.

The protein extracts (15 μg protein) diluted in sample buffer were resolved on 10% polyacrylamide gels, and transferred onto polyvinylidene difluoride membranes (Merck Millipore, Darmstadt, Germany). Membranes were blocked in phosphate saline buffer with 0.05% Tween-20 (PBS-T) containing 2% bovine serum albumin for 1 h at room temperature. Thereafter, membranes were incubated with primary antibodies overnight at 4°C. Blots were then washed with PBS-T and probed for 1 h at room temperature with the correspondent secondary antibodies conjugated with horseradish peroxidase. Finally, membranes were stripped with Tris/HCl buffer pH 2.3 containing beta-mercaptoethanol 0.1 M and re-probed with anti-beta-actin to perform loading controls. Immunoblot signals were detected using a chemiluminescence kit according to the manufacture’s instruction (Pierce ECL 2 Western Blotting Substrate; Thermo Fisher Scientific, MA, United States) (20). Density of all bands was determined by densitometry using Image Quant LAS 500 software (GE Healthcare, CA, United States). Relative protein expression data were normalized against β-actin level.

### Statistical Analysis

**FIGURE 2:**
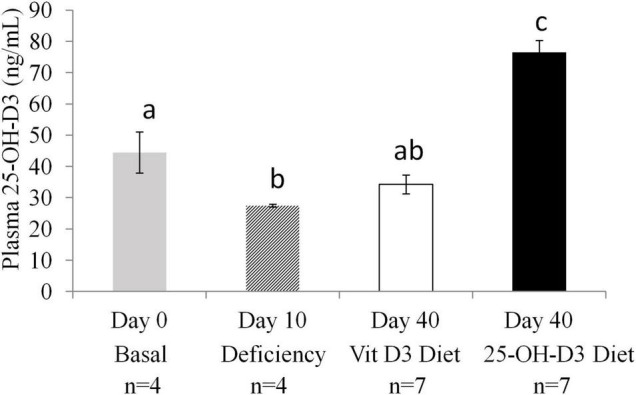
Plasma 25-OH-Vitamin D3 levels in non-pregnant animals at different time points: at study entry (Basal *n* = 4), after a deficiency diet (*n* = 4) and after 4 weeks of feeding with test diets containing 25-OH-D3 (experimental diet *n* = 7) or vitamin D3 (*n* = 7) before mating. ANOVA, *P* < 0.001. Values not sharing a common superscript letter are significantly different (*P* < 0.05).

Shapiro-Wilk normality test was used to check normal distribution of continuous variables. The results were expressed as mean ± standard error of the mean (SEM). The two experimental groups were compared using unpaired *t*-test analyses. ANOVA test was also applied for multiple testing followed by *post-hoc* Bonferroni in the comparisons of Figure 2. Pearson correlations were also performed. Chi^2^ analysis of qualitative data were also analyzed. The statistical analyses were evaluated by the SPSS^®^ 24.0 software package (IBM Corp., NY, United States). A *p*-value 0.05 was considered to be statistically significant.

## Results

Maternal, fetal, and placental weights were not significantly different between the groups fed 25-OH-D3 diet and the control group that was fed vitamin D3 (Table 1). The number of fetuses per dam was also similar between both diet groups. In addition, no differences were found in the abortion rate between both groups. There was also no difference in fertility rate. In summary, pregnancy-related outcomes are comparable between the study groups (Table 1).

**TABLE 1:** Animal characteristics and dietary intake.

	Experimental (*n* = 18)	Control (*n* = 16)	*P*
**Maternal weight (g)**			
At the start of the study	163.99 ± 3.08	169.02 ± 5.14	0.408
After deficiency (day 10)	192.68 ± 3.79	195.74 ± 4.50	0.604
At delivery	346.14 ± 8.13	355.69 ± 6.77	0.384
Dietary intake before pregnancy (g/d)	11.73 ± 0.31	12.12 ± 0.35	0.416
N° fetus	11.17 ± 1.01	12.81 ± 0.56	0.177
Fetal weight (g)	3.76 ± 0.19	3.34 ± 0.16	0.097
Placental weight (g)	0.56 ± 0.05	0.50 ± 0.01	0.202
Abortions per rat	0.28 ± 0.14	0.13 ± 0.09	0.361
No pregnant rats (n/%)*	2 (10%)	1 (6%)	0.647

*Media ± SEM or n (%). Significance level set at P < 0.05 by t-test.*

*Differences evaluated by Chi^2^.

The 10-day deprivation period resulted in significant decreases in plasma levels of 25-OH-D3 compared to the baseline level detected at the start of the study (Day 0) (Figure 2). The animals were split into two groups and were fed different diets for 4 weeks (Day 40). At Day 40, plasma levels of 25-OH-D3 were significantly higher in the 25-OH-D3 group than in the vitamin D3 control group. Furthermore, the level of 25-OH-D3 in the 25-OH-D3 group was higher than the baseline level detected at the study entry (Figure 2). In contrast, vitamin D3 group plasma 25-OH-D3 levels were not different from baseline levels. The data showed that 25-OH-D3 supplementation resulted in higher levels of 25-OH-D3 in plasma than vitamin D3 supplementation in non-pregnant animals.

**FIGURE 3:**
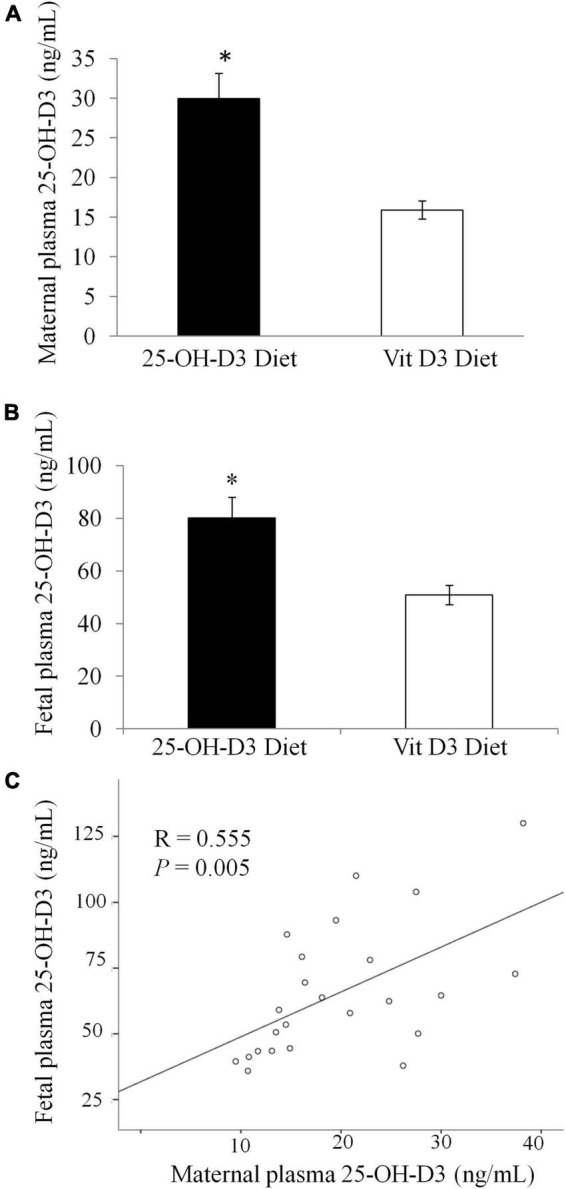
Concentration of 25-OH-Vitamin D3 in plasma at delivery in rats treated during pregnancy with 25-OH-D3 (experimental diet) or vitamin D3 control diet. **(A)** Concentration of 25-OH-Vitamin D3 in maternal plasma at delivery (*P* < 0.001). **(B)** 25-OH-Vitamin D3 in fetal plasma at delivery (*P* = 0.003). **(C)** Correlation between the levels of 25-OH-D3 in maternal and fetal plasma. *T*-test significant differences *P < 0.05.

At delivery, maternal plasma levels of 25-OH-D3 were significantly higher in the 25-OH-D3 group compared to those of the vitamin D3 group (Figure 3A). The maternal plasma concentration of 25-OH-D3 group was almost two times higher than that found in the vitamin D3 group. Fetal plasma 25-OH-D3 levels were also significantly higher in the 25-OH-D3 group compared to those of the vitamin D3 group (Figure 3B). The levels of fetal plasma 25-OH-D3 in the 25-OH-D3 group were about 1.6× higher than those detected in the vitamin D3 group (50 ng/mL in experimental group vs. 80 ng/mL in control). Thus, the results showed that 25-OH-D3 supplementation had higher potency in raising vitamin D status in both maternal and fetal plasma during pregnancy compared to vitamin D3 supplementation. There was a significant correlation between the levels of 25-OH-D3 in maternal and fetal plasma (*r* = 0.555, *P* = 0.005). 25-OH-D3 is the vitamin D metabolite that crosses the placenta. The ratio of fetal to maternal 25-OH-D3 concentrations at delivery was similar in all groups (25-OH-D3 fetal/maternal plasma: 3.51 ± 0.46 in the 25-OH-D3 group vs. 3.56 ± 0.13 in the vitamin D3 group, *p* = 0.908).

**FIGURE 4:**
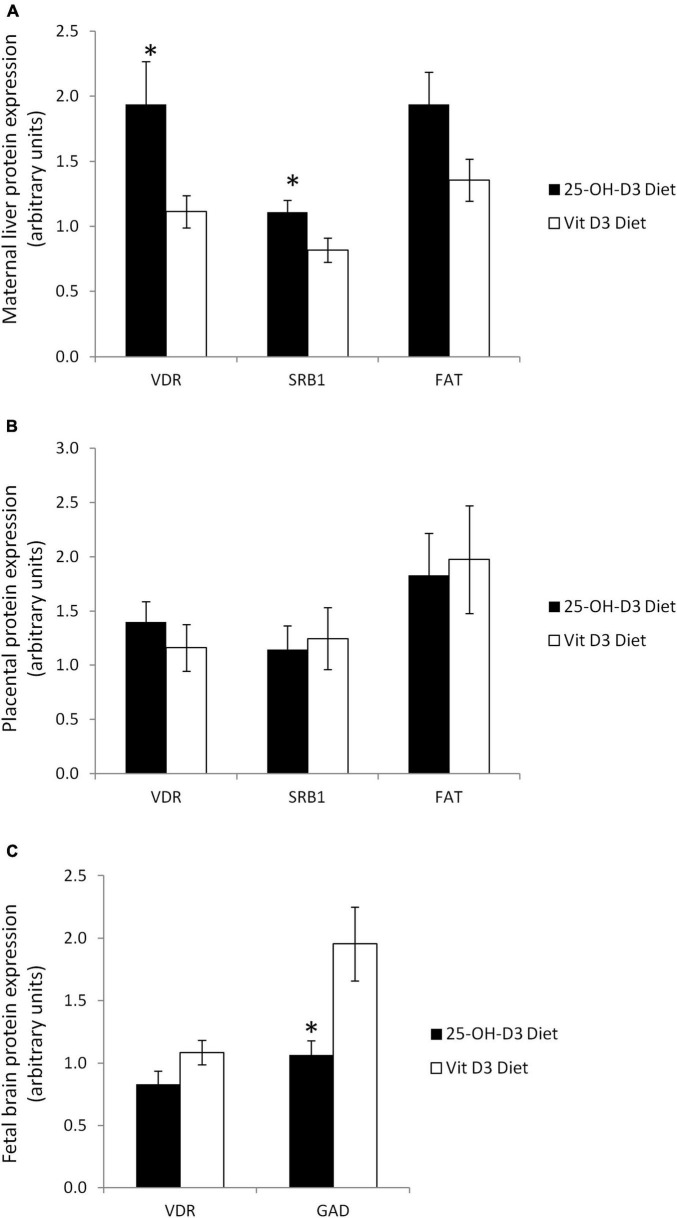
Vitamin D related proteins in several tissues at delivery in rats treated during pregnancy with 25-OH-D3 (experimental diet) or vitamin D3 (control diet). **(A)** Maternal liver: vitamin D receptor (VDR), fatty acid translocase (FAT/CD36) (*P* = 0.059), and Scavenger Receptor 1 (SR-B1); **(B)** placenta: VDR, FAT/CD36, and SR-B1; **(C)** fetal brain at delivery: VDR (*P* = 0.086) and glutamate decarboxylase (GAD). *T*-test significant differences *P < 0.05.

With regards to vitamin D metabolism, pregnant rat dams supplemented with 25-OH-D3 had significantly higher VDR hepatic protein levels compared to the vitamin D3 group (Figure 4A). The increase in protein levels were also observed for SR-B1 (*p* < 0.05) and FAT/CD36 (*p* = 0.059) (Figure 4A). The findings showed that the higher vitamin D levels in pregnant rat dams corresponded with higher expressions of vitamin D metabolism-related proteins, such as VDR which is a known receptor for 1,25-(OH)2-D3.

Interestingly, the protein levels for VDR, SR-B1, and FAT/CD36 in the placentas were similar in the two groups (Figure 4B). This result suggested that placental transfer of 25-OH-D3 from the mother to the fetus was not affected, highlighting the importance of achieving an optimal maternal 25-OH-D3 concentration for increasing fetal serum vitamin D status.

Due to the remarkable results observed in maternal liver, we decided to analyze VDR levels in fetal brain. Surprisingly, maternal 25-OH-D3 supplementation tended to decrease VDR expression in fetal brain (*p* = 0.086) although the differences were not statistically significant.” This is probably due to a negative feedback mechanism to protect the fetal brain (Figure 4C). In addition, we also detected lower expression of GAD67, which is an established marker of GABAergic neurons, in the fetal brain by maternal 25-OH-D3 supplementation during pregnancy (Figure 4C). These results suggested that the impacts caused by the increase in vitamin D status in the fetuses were systemic.

## Discussion

We report for the first time during pregnancy that supplementation with 25-OH-D3 dramatically increased plasma 25-OH-D3 in both the dams and fetuses compared to D3 with important perturbations in the levels of vitamin D relevant proteins in both mother and fetus. For this study, we did not adjust the diets for vitamin D activity since the conversion rate for 25-OH-D3 remains uncertain. Vitamin D3 has a recognized biological activity of 40 IU per microgram. This conversion factor has been used to achieve 1,000 IU/kg in both the vitamin D and 25-OH-D3 diets. Nevertheless, it should be noted that commercial hydroferol (25-OH-D3) has been reported to be 60 IU per microgram. Therefore, there is a possibility that 25-OH-D3 conversion factor should be 60 IU per microgram or more instead of 40 IU per microgram. This could also explain the results obtained by this study.

Our study showed that 25-OH-D3 supplementation increased the level of 25-OH-D3 in both maternal and fetal serum two times more effectively than supplementation with a matching level of vitamin D. The higher availability in fetal 25-OH-D3 occurred without differences in VDR, FAT/CD36, or SR-B1 protein expressions in placental tissue. The results on the relationship between placental VDR and 25-OH-De are scarce and contradictory. In mice, placental VDR expression was significantly up-regulated in vitamin D3-pretreated animals supporting anti-inflammatory effects against lipopolysaccharide in the placenta (21). However, in adolescents, placental VDR expression was inversely associated with neonatal 25(OH)D (*P* = 0.012) and maternal 25(OH)D (*P* = 0.080) while positively with neonatal 1,25(OH)2D (*P* = 0.006) (22). In gestational diabetes, low vitamin D was reported in serum while higher levels of placental VDR; in fact, low serum levels could even up-regulate the placenta VDR gene expression *via* negative feedback regulation, such that the increase in the bioavailability of vitamin D might compensates for the deficiency (23). Placenta is a key organ of transfer for 25-OH-D3 that even expresses the enzyme 1-α-hydroxylase, to synthesize 1,25-(OH)2-D3 and hence to regulate inflammatory processes. For this reason, the protein levels of VDR in placenta may differ to those in maternal liver. In addition, no changes in pregnancy outcomes or fetal weight were found between the groups in the present study.

Although 25-OH-D3 or calcifediol has been widely used for dietary supplementation of vitamin D, it is not approved for use in pregnant women as there is lack of safety data from randomized controlled trials. An additional problem with the use of 25-OH-D3 is that vitamin D3 and calcifediol are not equipotent (6) and there is no consensus on the conversion factor that should be used for 25-OH-D3 to calculate vitamin D activity (IU units). Conversion-factor estimates of 1.4 and 5-fold-increase arose in two intervention studies with patients who required vitamin D treatment (2, 24). A study conducted in winter in older adults reported that each microgram of oral 25-OH-D3 was about five times more effective in raising serum 25-OH-D3 in older adults than an equivalent amount of vitamin D3 (2); oral supplementation with 25 μg calcifediol reached 134.6 ± 26 nmol/L 25-OH-D3 in serum compared to 69.0 ± 8.7 nmol/L using the same dose of vitamin D3 (2). In contrast, Bischoff-Ferrari et al. (7) showed that in healthy postmenopausal women (*n* = 10 women per group), oral supplementation of 25-OH-D3 increased plasma 25-OH-D3 levels three times more effectively than a matched dose of vitamin D3. 25-OH-D3 supplementation rapidly and safely elevated serum 25-OH-D3 concentrations in a dose-dependent manner to improve vitamin D status in different populations (3); a daily dose of 10 mg of 25-OH-D3 maintained serum 25-OH-D3 concentrations between 75 and 100 nmol/L (3). However, data that help to define the conversion factor for 25-OH-D3 during pregnancy are lacking. The data generated by this study will provide another piece of information to help define the intake of vitamin D and circulating level of 25-OH-D3 in pregnant women that is adequate to improve fetal development and prevent maternal complications.

In the present study, dietary 25-OH-D3 significantly increased VDR protein in maternal liver. We also observed 25-OH-D3-mediated increases in maternal liver levels of the fatty acid transporter FAT/CD36 and the cholesterol carrier SR-B1. These findings suggest changes in the transport of lipophilic nutrients such as vitamin D in this organ. Higher FAT and SR-B1 protein levels in the maternal liver might result in higher uptake of 25-OH-D3, which may in turn increase its activity. Since the actions of vitamin D are mediated by VDR that binds 1,25(OH)2D3, this could also support higher active form of vitamin D in the maternal liver. Recently, Kiourtzidis et al. (25) reported in mice deficient in SR-B1 (Srb1−/−) or in CD36 (Cd36−/−) that received triple-deuterated vitamin D3 (vitamin D3-d3), they had significantly lower levels of 25-OH-D3-d3 in serum and tissues than in wild type animals; this study also confirmed that SR-B1 is not only crucial for the hepatic uptake of HDL cholesterol but also for the uptake of vitamin D into the liver to synthesize 25-(OH)-D, the primary biomarker of vitamin D status (25).

Interestingly, low serum levels of 25-(OH)-D have been observed in patients suffering obesity or non-alcoholic liver diseases compared to those of healthy subjects (26–28). CD36 levels are increased in several studies of NAFLD where they correlate with hepatic liver content (29, 30) but not in all (12). Despite higher SR-B1 or FAT/CD36 could favor higher vitamin D tissue uptake in these patients, the large amount of fat stored in tissue might reduce their final levels of 25-OH-D3 in serum. Whether vitamin D supplementation improves NAFLD has remained controversial in clinical trials (31–33). In mice, vitamin D supplementation alleviated NAFLD by activating VDR, whereas hepatic-specific knockout of VDR abolished the ameliorative effects of vitamin D on NAFLD (34). The higher levels on liver VDR by supplementation with 25-OH-D3 vs. vitamin D3 in the present study could be of major interest.

Placenta is a key organ that mediates nutrient transfer. It is important to note that 1,25-(OH)-2D does not practically cross the placental tissue, while its inactive precursor 25-(OH)-D readily crosses the tissue to the fetal compartment (8, 9). In the present study, the administration of 25-OH-D3 did not change the expression of FAT, SR-B1 in the placenta by the type of supplement. The lipid transport in the placenta and the cholesterol uptake/efflux is different than in the liver which could explain the differences between tissues. Nevertheless, this is the first study on the effect of the different types of vitamin D supplements in these placental carriers.

Vitamin D receptor is the single known regulatory mediator of hormonal 1,25-(OH)2-D3 in higher vertebrates (10). It acts in the nucleus of vitamin D target cells to regulate the expression of genes whose products control diverse cell type-specific biological functions that includes mineral homeostasis. However, as VDR expression emerged in other tissues, it became clear that vitamin D action in many cellular targets was unrelated to mineral regulation, suggesting additional vitamin D hormone functions (35). One surprising finding here is the trend of down-regulation of VDR in the fetal brain (*p* = 0.086) in the 25-OH-D3 group when compared to the vitamin D3 control group. Despite this result, was not statistically significant this down regulation could be a self-protection mechanism triggered by the high vitamin D level in the circulation (Figure 3C). In addition, 25-OH-D3 supplementation reduced the expression of glutamate decarboxylase GAD67, one of the GABAergic neuronal markers. Glutamate decarboxylase (GAD) is localized only in presynaptic terminals of GABAergic inhibitory neurons. There are two common forms of GAD-GAD65 and GAD67. These isoforms are encoded by independent genes with different subcellular localizations. GAD67 is localized in the cell soma of inhibitory neurons. GAD67 knocked-out mice have reduced GABA levels throughout the brain, a reduction in GAD activity, and severe cleft palate which leads to death within 24 h after birth (36). In this study, we found that the expression of VDR in fetal brain was positively associated with GAD67 (*R* = 0.391, *P* = 0.033). The relationship between Vitamin D and GABAergic neurons had been reported previously. GABA-Aα4 (37) and GABA B receptor 1 (38) expression was decreased in vitamin D deficient animals. However, some other studies have reported no difference in GABA transmission (39, 40), although the discrepancies between studies could be due to differences in the brain regions analyzed. We found changes in fetal vitamin D metabolism, beyond the serum levels of 25-OH-D3, that should be investigated in the fetus since it is in active neurodevelopment.

There is consensus that adequate vitamin D is necessary during pregnancy for maintaining both maternal calcium homeostasis and fetal bone development. There are also on-going discussions about the potential effects of vitamin D levels on pregnancy outcomes, such as preterm birth, gestational diabetes, preeclampsia risk, and also on children’s long-term health outcomes such as asthma and neurodevelopment (41–43). Hajhashime et al. (44) showed that direct sunlight exposure for 30 min daily (30% of body surface area) for 10 weeks can provide 25-OH-D3 levels of almost 20 ng/mL (up from 15.09 ng/mL) in the plasma of pregnant women with vitamin D deficiency. However, the same study also showed that dietary supplementation of vitamin D3 at 4,000 IU per day for 10 weeks increased 25-OH-D3 plasma level to 31.27 ng/mL (up from 15.95 ng/mL), which is significantly higher than the level achieved by sun exposure. On a side note, increase in sun exposure is associated with increase in cancer risk. Therefore, dietary supplementation of Vitamin D is a much more feasible intervention strategy than sun exposure for addressing vitamin D deficiency issues in pregnant women.

25-OH-D3 supplementation in pregnant women is of major interest because it could be likely achieve desirable maternal and fetal blood levels at lower doses than Vitamin D3. In fact, some endocrinologists are using it in pregnant women even though it is not approved for use in pregnancy due to the lack of safety data. Therefore, it is important to study the bioavailability of the different forms of vitamin D in during pregnancy. It is not feasible to extrapolate from other study populations due to the physiological changes and hemodilution that occur during pregnancy.

In conclusion, 25-OH-D3 is more potent than Vitamin D3 in raising the vitamin D status in pregnant rats Supplementation with 25-OH-D3 increased maternal and fetal 25-OH-D3 plasma concentrations by nearly two times compared to vitamin D3. 25-OH-D3 supplementation also increased VDR levels and some lipid carriers in maternal liver as SR-B1 and FAT/CD36, but this increase was not found in the placenta. In contrast, maternal 25-OH-D3 decreased fetal brain VDR and GAD. Thus, supplemental 25-OH-D3 improved maternal and fetal vitamin D status better than vitamin D3. Its effects on fetal tissues should be further explored in future studies.

## Data Availability Statement

The datasets generated for this study are available on request. The raw data supporting the conclusions of this article will be made available by the authors, without undue reservation.

## Ethics Statement

The animal study was reviewed and approved by the Institutional Animal Care and Use Committee of the University of Murcia (N° A13180105).

## Author Contributions

AG supervised the animal experiment, performed vitamin D analysis, and contributed to wrote the manuscript. MS-C performed Western blot analysis. EL conceived the study, designed and conducted the research, and had primary responsibility for the final content. AB, RR, and JC revised the manuscript. All authors have read and approved the final version of the manuscript.

## Conflict of Interest

RR, JC, and MK are employees at Abbott Nutrition S.L. The remaining authors declare that the research was conducted in the absence of any commercial or financial relationships that could be construed as a potential conflict of interest.

## Publisher’s Note

All claims expressed in this article are solely those of the authors and do not necessarily represent those of their affiliated organizations, or those of the publisher, the editors and the reviewers. Any product that may be evaluated in this article, or claim that may be made by its manufacturer, is not guaranteed or endorsed by the publisher.

## Abbreviations

25-OH-D3: hydroferol
FAT/CD36: fatty acid translocase
GAD: glutamate decarboxylase
GAD67: glutamic acid decarboxylase 67
PBS-T: phosphate saline buffer with 0.05% Tween-20
SEM: standard error of the mean
SR-B1: scavenger receptor class B type-1
VDR: vitamin D receptor
Vitamin D3: cholecalciferol.
